# Exploring Technical Features to Enhance Control in Videoconferencing Psychotherapy: Quantitative Study on Clinicians’ Perspectives

**DOI:** 10.2196/66904

**Published:** 2025-04-01

**Authors:** Francesco Cataldo, Shanton Chang, Antonette Mendoza, George Buchanan, Nicholas Van Dam

**Affiliations:** 1 School of Computing and Information System University of Melbourne Melbourne Australia; 2 School of Computing Technologies Royal Melbourne Institute of Technology University Melbourne Australia; 3 Melbourne School of Psychological Sciences University of Melbourne Melbourne Australia

**Keywords:** videoconference psychotherapy, control, therapeutic relationship, therapeutic alliance, videoconference technologies, technological features, video, telepsychiatry, videoconferencing, psychotherapy, mental health, mental, therapy, therapist, videoconference, platform, psychology, psychologist

## Abstract

**Background:**

The COVID-19 pandemic required psychologists and other mental health professionals to use videoconferencing platforms. Previous research has highlighted therapists’ hesitation toward adopting the medium since they find it hard to establish control over videoconferencing psychotherapy (VCP). An earlier study provided a set of potential features that may help enhance psychologists’ control in their videoconference sessions, such as screen control functionality, emergency call functionality, eye contact functionality, zooming in and out functionality, and an interactive interface with other apps and software.

**Objective:**

This study aims to investigate whether introducing technical features might improve clinicians’ control over their video sessions. Additionally, it seeks to understand the role of the video in therapists’ VCP experience from a technical and relationship point of view.

**Methods:**

A total of 121 mental health professionals responded to the survey, but only 86 participants provided complete data. Exploratory Factor Analysis was used to scrutinize the data collected. A total of three factors were identified: (1) “challenges in providing VCP,” (2) “features to enhance the therapeutic relationship,” and (3) “enhancing control.” Path analysis was used to observe the relationship between factors on their own and with adjustment to participants’ areas of expertise and year in practice.

**Results:**

This study highlighted a relationship between the three identified factors. It was found that introducing certain features reduced therapists' challenges in the provision of VCP. Moreover, the additional features provided therapists with enhanced control over their VCP sessions. A path analysis was conducted to investigate the relationships between the factors loaded. The results of the analysis revealed a significant relationship between “challenges in VCP” and “features to enhance the therapeutic relationship” (adjusted beta [Adjβ]=–0.54, 95% CI 0.29-0.79; *P*<.001). Additionally, a significant positive relationship was found between “features to enhance the therapeutic relationship” and “enhancing control” (Adjβ=0.25, 95% CI 0.15-0.35; *P*<.001). Furthermore, there was an indirect effect of “challenges in providing VCP” on “enhancing control” (Adjβ=0.13, 95% CI 0.05-0.22; *P*=.001) mediated by “features to enhance TR.” The analysis identified the factor “features to enhance TR” (effect size=0.25) as key for improving clinicians’ performance and control.

**Conclusions:**

This study demonstrates that technology may help improve therapists’ VCP experiences by implementing features that respond to their need for enhanced control. By augmenting therapists’ control, clinicians can effectively serve their patients and facilitate successful therapy outcomes. Moreover, this study confirms the video as a third agent that prevents therapists from affecting clients’ reality due to technical and relational limits. Additionally, this study supports the general system theory, which allowed for the incorporation of video in our exploration and helped explain its agency in VCP.

## Introduction

The COVID-19 pandemic required psychologists worldwide to discontinue face-to-face (FTF) psychotherapy and deliver psychotherapy sessions through videoconferencing technologies (VCTs) [1]. Although necessary, social distancing revolutionized the psychological and social dimensions of private and public life [2], which worsened the onset of psychological symptoms, such as anxiety and posttraumatic stress [3-6]. The aggravation of such symptoms during social distancing resulted in increased videoconferencing psychotherapy (VCP) requests [7,8] and reinforced the perspective of VCP as a tool that facilitates psychotherapy for people living remotely [9-11]. Moreover, data show that VCP adoption is likely to continue increasing [12-14] alongside the volume of publications related to web-based psychotherapy services [13,15-19]. These factors, along with the growing number of VCP advocates compared with past pandemic trends [16-19], have made VCP a relevant phenomenon for psychologists, patients, and researchers worldwide. Therefore, investigating VCP becomes paramount for a conscious implementation of VCTs for therapeutic purposes.

Prior to COVID-19, VCP was already being adopted to overcome geographical barriers [20,21], and research proved its effectiveness [22-24] but not its interchangeability with FTF treatment [25-28]. Prepandemic studies on patient perspectives on VCP showed that patients rated their VCP experience positively [29-31], while psychologists remained skeptical toward the medium [32]. Following the pandemic, literature from before and after COVID-19 both identify analogous benefits and limitations of VCT adoption [2]. More precisely, VCP improves psychotherapy accessibility by providing a flexible and cost-effective alternative to FTF therapy [33-35], but it also gives rise to technical and relational concerns from psychologists’ perspectives [32]. Certainly, COVID-19 forced psychologists to be more exposed to VCTs, reducing some of their technical concerns over time. However, many psychologists are still resistant to the medium because of technological constraints [10,32] and feelings of inadequacy they experience while managing VCTs [36].

From a technical standpoint, numerous therapists remain fearful that video glitches, inadequate internet literacy, granting confidentiality online [37], and impaired nonverbal communication might lead to miscommunications and a high proportion of dropouts [2,10,38], hindering therapeutic success [39]. VCTs certainly provide users with an environment with fewer physical or visual cues [40], which may in turn result in frustrating and exhausting interactions [41]. Technical delays and camera mediation may also compromise patient-therapist communication and diminish chances for synchronous verbal responses, body language, and eye gaze detection [41].

Moreover, the literature detects a correlation between the issues derived from technical failures related to VCT and therapy outcomes [32,42-45], suggesting that the complex management of the virtual setting, trust breakability, and inadequate gathering of visual cues (ie, poor detection of body language and restricted eye contact) may affect the success of the therapeutic intervention [9,10,14,46] since it compromises a therapist’s understanding of their patient’s psychological state [47].

Therapists’ concerns about the management of VCTs and VCP were analyzed in a previous study [10] where a varied sample of therapists responded to a semistructured interview based on their VCP experience. The analysis of the findings resulted in the individuation of therapists’ poor VCP control (the term “control” here is meant as a system where each participant is able to control or influence the behavior of the other [48]) as the core issues in their VCP sessions and in the formulation of a set of technical features that, according to the sample interviewed, might address therapists’ limited control over VCP. The features include screen control functionality, emergency call functionality, eye contact functionality, zooming in and out functionality, and an interactive interface with other apps and software. Furthermore, the therapists interviewed suggested that, besides strengthening their control over the therapy, these features might ease emotional and body language communication and reinforce relational patterns. Additionally, through the set of features given, researchers developed the VCP-IPO (input-process-output) conceptual model to investigate whether the suggested technical requirements (input) might be predictive of the correct fulfillment of the therapist’s processes in VCP (eg, trust development, emotional communication, and engagement with patients).

An impairment of the aforementioned processes may lead therapists to experience limited control over their VCP sessions, affecting the relational aspects of the psychotherapy service. Indeed, therapists’ primary concern remains their control over the therapist-patient relationship during therapy [49], which includes practically managing VCTs [10,32] and fostering a satisfactory relationship with patients [7]. Another study [47] had similar results where therapists experienced reduced control in VCP compared to FTF sessions due to the impossibility of controlling the patients’ environment and intervening if crises did occur. These VCP challenges affect therapists’ confidence and the gratification they obtain from delivering VCP. As a result, psychologists fear that VCP may detract from therapy effectiveness compared with FTF sessions [19,39], and they are reluctant to consider VCP an equally effective alternative to FTF psychotherapy [9,10,14,50-52]. Therapists also experience reservations about the impact of VCP on the therapeutic alliance (TA) and the therapeutic relationship (TR) [53]. TA refers to the patient’s and therapist’s mutual engagement in the therapy process [54], whereas TR concerns the attitudes and feelings experienced by the therapist and the patient toward each other [55]. TA and TR contribute enormously to the treatment success [54]. In fact, weak TA and TR might result in fragile trust and ineffective communication [56,57], negatively affecting the outcome of the therapy [58-61].

Considering the aforementioned technical and relational concerns, it is expected that VCT platforms may develop additional features to support psychologists in their VCP experience. In 2021, IT organizations invested heavily in cloud-based technologies and digital collaboration tools to support professionals working from home [11]. Nevertheless, it seems that the existing technology is not satisfactory for clinicians who require specific improvements to support their work and strengthen their control over VCP. Consequently, it is crucial to address psychologists’ need for control and understand whether the proposed additional technical features may respond to this need.

In the absence of previous studies exploring the relationship between the technical features suggested (screen control functionality, emergency call functionality, eye contact functionality, zooming in and out functionality, and interactive interface with other apps and software) and improved VCP control, the guiding research question to be addressed in this study is: Can therapists enhance their control over VCP by incorporating additional technical features?

It is important to clarify that this study explores VCP issues that therapists deem as affecting their control over VCP regardless of the therapeutic approach applied. Certainly, different therapeutic approaches may involve distinctive issues and technical needs. A study conducted in 2021 suggests that Cognitive Behavioral Therapy is suitable for VCP due to its standardized methodology and minor reliance on the psychologist-patient relationship compared with other approaches [62]. According to Armfield et al [63], VCP may be the best fit for low-risk patients and situational crises such as the COVID-19 pandemic. Additionally, VCP may not be the best option for some interventions, such as intentional silence (to enhance a patient’s exploration [64]) and the empty-chair dialogue, for therapists who may not like working by video for personal factors, or for clinicians who may struggle to apply specific techniques over video [47].

To address the goal, a survey was developed a survey to determine whether the aforementioned suggested technical features can help psychologists boost control over their VCP experiences.

## Methods

### Recruitment

A quantitative analysis was performed. According to Tabachnik and Fidell (1998), a minimum of five participants per question (5:1 ratio) is necessary. The survey (Table S1 in Multimedia Appendix 1) included 24 questions, thus requiring a minimum of 121 participants to conduct the study. The sample was recruited using a convenience sampling approach by author FC (who is a PhD candidate and clinical psychologist in Italia and Australia) via LinkedIn, professional websites, official psychologists, and other mental health associations from October 16, 2022, to February 2, 2023. Using author FC’s university email address, Practitioners were emailed the plain language statement and the link to the survey. Participants could access the web-based survey at any time and from any location (no restriction or time frame was provided) using their mobile phone or a computer/laptop. No reminders were sent, and no technical difficulties were recorded. The language used in the survey was English.

**Figure 1:**
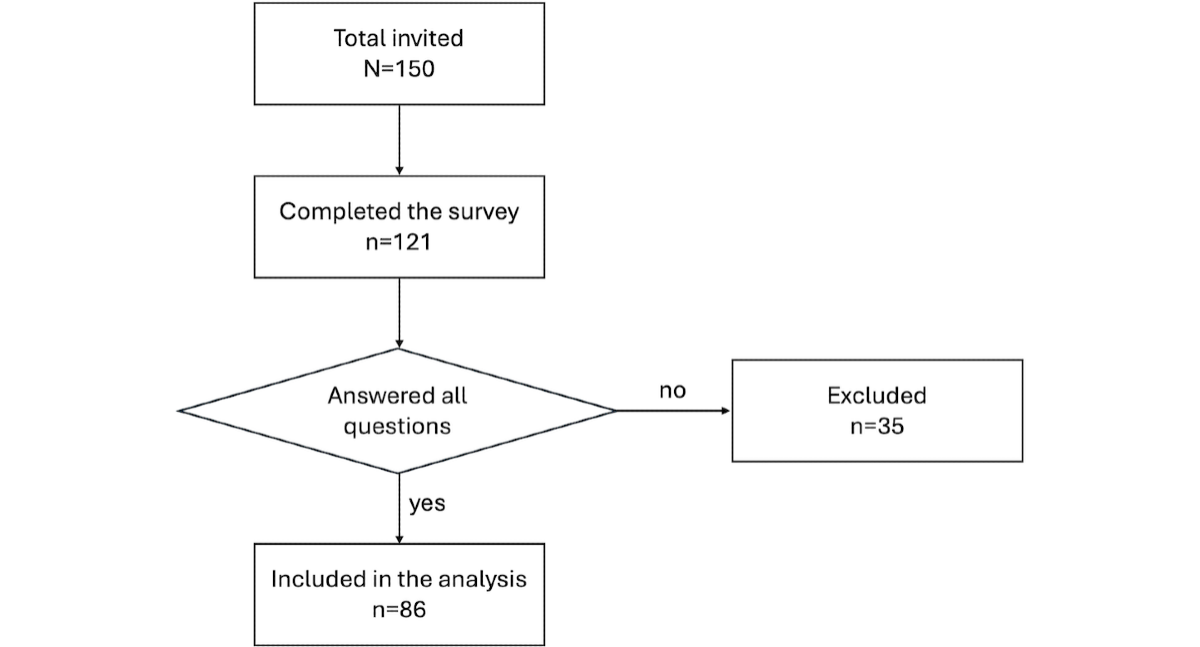
Flowchart: sample of respondents included in our analysis.

Data collection started on October 19, 2022, and follow-up was not performed due to the cross-sectional nature of the survey. Unfortunately, from the 121 practitioners (Figure 1) recruited into this study, only 35 returned surveys with a clear pattern of missing data. All data were exported from Qualtrics software (Qualtrics Inc) to Excel (Microsoft Corp) to identify patterns of missing data. A comparison of the analysis with and without cases of missing data was conducted. Therefore, the responses of participants with missing data were excluded from the analysis, and there was no difference between including or excluding them in our results. As a result, the final sample included in the analysis comprised 86 participants, among whom 70 (81.4%) were psychologists, 6 (7%) were psychotherapists, and 10 (11.6%) were other mental health specialists. The participants had a median of 6 (IQR 4-6) years of practical experience. The participants were enrolled on a voluntary basis, and they were free to withdraw from participation at any time.

### Ethical Considerations

This study was approved by the University of Melbourne ethics committee (2022-13377-32266-8) on October 7, 2022. Participants provided informed consent after learning about the theme and purpose of the survey, together with the data collection process and their rights as respondents (plain language statement). Furthermore, they were informed that the data would be collected anonymously and personal information would be collected and stored in the survey. Participants were not reimbursed, as they completed the study on an entirely voluntary basis.

### Overview

Building on prior qualitative research (Cataldo, 2023) and literature, a closed, anonymous web-based survey was developed and emailed, restricted to mental health practitioners only. The survey comprised 27 randomized items on a 5-point Likert scale, with responses ranging from strongly agree to strongly disagree. The web-based survey did not apply adaptive questioning, and the same questions were submitted to all participants. The order of the questions was the same for each participant: 8 pages containing 3 questions each, 1 page with 2 questions, and the final page with the final question. Mandatory responses were applied, and respondents could not go to the previous page and modify their answers. Moreover, no tracking measures or other techniques were conducted to prevent multiple entries; consequently, the analysis was conducted based on the responses received. Although this approach enhanced privacy, it implied that duplicate answers could not be recognized.

Nevertheless, the controlled survey distribution relieved the risk, which was likely negligible. The participation rate was 71% (86/121), while the completion rate was 78%. The questionnaire was pretested with a small number of participants (n=6, 5%) to ensure the items were clear and coherent. The items did not gather demographic details such as sex and age; instead, the focus was on the practitioners' areas of expertise and years of experience. The development of the survey required an extensive study of the literature [32] and a search for previous questionnaires exploring similar and identical topics. Unfortunately, no studies investigating psychologists’ technological needs were found; therefore, most of the items mirror the technical features listed in the findings section of the previous study [10]. Furthermore, the choice of using a Likert scale followed common practice in studies capturing deep-seated descriptions [65]. The survey was built in Qualtrics, and the link was distributed to all study participants by email.

### Data Analytics

Descriptive statistics were used to summarize survey responses, and the results were reported as median (IQR). The correlation between items was assessed using Spearman correlation with Bonferroni correction for multiplicity. A very strong correlation was considered to be greater than 0.8, a moderate correlation between 0.4 and 0.8, a mild correlation between 0.2 and 0.4, and a weak correlation to be less than 0.2.

Exploratory factor analysis (EFA) was conducted to test the internal consistency and underlying factors that could describe the relationships among the items [66]. The number of factors identified was selected based on eigenvalue, as there was no underlying hypothesis on the number of factors. Moreover, a Kaiser-Meyer-Olkin (KMO) test was conducted to assess the suitability of the data. The Bartlett test of sphericity was also performed to evaluate whether the variables were related and appropriate for EFA, ultimately aiming to explore whether there was redundancy between variables that could be summarized into factors.

Following the EFA, total scores for each factor were calculated as the total sum of items included in each factor. Path analysis was used to further examine the relationship between factors on their own and with adjustment to participants’ areas of expertise and year in practice.

Path analysis is a set of regression models (a subset of the structured equation model) used to evaluate the causal directional relationship between variables. This method allowed to examine the direct and indirect effect of the variables on the outcomes and is very sensitive to the model specification. Moreover, this method is particularly used in testing well-specified theories that clearly define what factors need to be included in the model and what the possible relationships are. The analysis usually includes a path diagram that depicts the nature of the relationship between variables and outcomes [67]. SPSS Statistics for Windows (version 25.0; IBM Corp) and the open-source software Jamovi (for path analysis) were used for data processing and analysis.

## Results

### Correlation Analysis

The results of the correlation analysis are reported in Table S2 in Multimedia Appendix 2. This table shows the extent to which two variables were linearly related. Due to the moderate correlation between some items, oblique rotation was used during the factor analysis.

### EFA Results

The EFA was conducted in a subsample of 86 mental health workers who completed the survey. The KMO score was 0.97 and the Bartlett test of sphericity was significant (*P*<.001), indicating that the data were suitable for the EFA.

**Figure 2:**
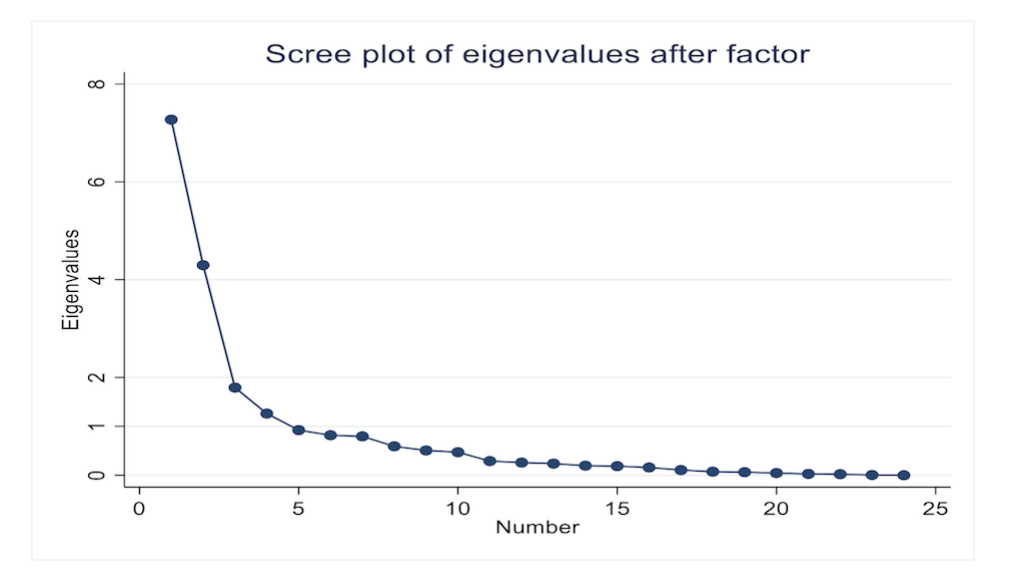
Scree plot of the 4 factors loaded.

Based on the eigenvalue (Table 1), 3 factors needed to be considered, while the Scree plot (Figure 2) showed 4 factors. However, attention was focused on reporting the 3-factor loading (Table 2) since the results obtained for the fourth factor were too weak. The fourth factor was formed by only two items that were not significant, leading to a focus on the 3-factor loading.

In accordance with Field [68], factor loadings lower than 0.3 were suppressed and only focused on stable scores, particularly scores higher than 0.4 [69]. The total score for each factor was calculated, and the results are reported in Table 2.

No direct relationship was observed between “challenges in providing VCP” and “enhancing control” (*r*=0.16; *P=*.15). However, the factor “features to enhance TR” was moderately correlated with “enhancing control” (*r*=0.46; *P*<.001) and “challenges in providing VCP” (*r*=0.44; *P*<.001).

**Table 1:** Eigenvalue of the 3 factors.

Factor	Variance	Difference	Proportion	Cumulative
Factor 1	5.67497	1.35003	0.4557	0.4557
Factor 2	4.32493	1.87087	0.3473	0.8030
Factor 3	2.45406	—^a^	0.1971	1.0000

^a^Not available.

**Table 2:** The 3 factors loaded.

Item			Challenges in providing VCP^a^		Features to enhance TR^b^		Enhancing control
**Attitude toward video session**					—^c^		—
	I feel the video hinders my psychotherapy sessions	0.89					
	I find it difficult to build a therapeutic relationship with my clients by video	0.85					
	I believe it is hard to connect emotionally with my clients through video	0.85					
	I struggle to communicate by video with my client	0.82					
	I struggle to build trust with my clients by video	0.79					
	I feel I have less control over my therapeutic relationship via video rather than face to face	0.76					
	My emotional and cognitive preparation before sessions varies depending on whether the encounter is online or face-to-face	0.47					
	I think I will keep using videoconference platforms to treat my clients in the future	–0.74					
	My attitude toward videoconference psychotherapy is positively changing	–0.63					
	I would use a phone call before the first video session to increase trust with my clients						
	I perceive the monitor as an additional member of the therapeutic interaction						
**Platform engagement features**			—				—
	Zooming in/out (focus on clients’ facial expressions/whole body) would allow me to understand clients’ emotions			0.77			
	To reduce my cognitive and emotional load I need a platform that allows me to focus on clients’ facial expressions (zooming in) and capture the whole body (zooming out)			0.76			
	I feel that zooming in/out (focus on clients’ facial expressions/whole body) would augment my level of engagement with clients			0.73			
	My cognitive and emotional load would be reduced if I could maintain better eye contact with clients during video sessions			0.72			
	The eye contact functionality (establishing-maintaining eye contact) would heighten my chances to empathize with clients zooming in/out			0.60			
	I would improve trust if I could establish and maintain better eye contact with my clients during video sessions			0.59			
	I feel I need to establish eye contact to reinforce my engagement with clients during my video sessions			0.50			
	My fatigue would be reduced if my telehealth platform enabled simultaneous interaction with other apps and software			0.55			
**Activity control**			—		—		
	Limiting my clients’ online activities (e-mails, notifications, browsing online, etc) during our sessions would give me more control over our relationship					0.82	
	The technical ability to limit clients’ online activities during sessions would boost my sense of presence with clients					0.78	
	Limiting my clients’ online activities (emails, notifications, browsing online, etc) during our sessions would support me in enhancing my engagement with clients					0.70	
Scores for each factor, mean (SD)			20.6 (5.3)		25.6 (6.9)		9.3 (3.3)

^a^VCP: videoconference psychotherapy.

^b^TR: therapeutic relationship.

^c^Not applicable.

**Figure 3:**
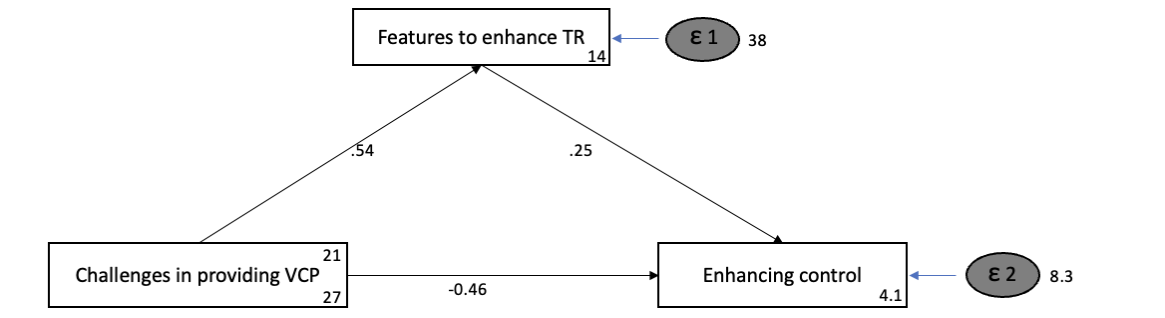
Path analysis of the 3 factors loaded. TR: therapeutic relationship. VCP: videoconference psychotherapy.

The results of the path analysis, both crude (Figure 3) and adjusted for participants’ areas of expertise and years in practice, also show no direct effect of “challenges in providing VCP” and “enhancing control” (crude adjusted beta [Adjβ]=–0.05, 95% CI –0.17 to 0.08; *P=*.48 vs adjusted Adjβ=–0.05, 95% CI –0.18 to 0.08; *P*=.46). However, the results showed a significant relationship between “challenges in VCP” and “features to enhance TR” (crude Adjβ=–0.54, 95% CI 0.29-0.79; *P<.*001 vs adjusted Adjβ=–0.56, 95% CI 0.30-0.81; *P*<.001) and “features to enhance TR” and “enhancing control” (crude and adjusted: Adjβ=0.25, 95% CI 0.15-0.35; *P*<.001). This also indicates that there was an indirect effect of “challenges in providing VCP” on “enhancing control” (crude and adjusted: Adjβ=0.13, 95% CI 0.05-0.22; *P=*.001), mediated by “features.” The results did not highlight any substantial difference between the crude and adjusted results.

The data showed a positive relationship (effect size=0.54) between “challenges in providing VCP” and “features to enhance TR”, where “features” was a medium to “enhancing control” (effect size=0.25). This implies that the number of challenges clinicians deal with is directly proportional to the variety and the number of features needed. Additionally, a lack of a direct relationship was noted between “challenges in providing VCP” and “enhancing control.”

## Discussion

### Exploration of the 3 Loaded Factors

This study aimed to ascertain whether implementing technical features could help enhance therapists’ control over their VCP sessions. To address the aim, 121 mental health practitioners were recruited to participate in the study. Several questionnaires were returned presenting a pattern of missing data; thus, the final sample included 86 participants. The factor analysis loaded three main factors: (1) “challenges in providing VCP,” (2) “features to enhance TR,” and (3) “enhancing control.” The first factor showed how therapists struggle to conduct VCP, build trust, foster a TR to connect emotionally with their clients, and communicate by video. The second factor concerned the technical features that might improve therapists’ experience with VCP. More precisely, therapists supported the introduction of functionalities that would help them establish better eye contact, enhance presence, and improve nonverbal communication by zooming in/out. The third factor highlighted therapists’ need to strengthen their control over VCP. The factors outlined the therapists’ need for technical features that can help boost their control over their VCP sessions. These technical measures could enhance clinical work and address clinicians’ concerns about the management of VCP.

The limited availability of analogous studies makes it difficult to compare the results with prior work. This study analyzes the relationship among the factors identified and provides directions for possible VCT enhancements [70]. As mentioned earlier in this paper, in a previous study [10], psychologists suggested a set of features that could help boost their experience of control in their VCP sessions. This perceived lack of control refers to technical and relational aspects of their VCP experience.

Interestingly, the analysis identified the factor “features to enhance TR” (effect size=0.25) as a key factor for improving clinicians’ performance and control. The path analysis showed how “features to enhance TR” (second factor loaded) alleviated therapists’ “challenges in providing VCP” (first factor loaded), and this had a roundabout impact on the third factor loaded—“enhancing control.” Moreover, there was no direct relationship between “challenges in providing VCP” and “enhancing control,” possibly because control (a multidimensional factor in our analysis) represented the therapists’ outcome experience; this would make it less directly influenced by “challenges in providing VCP”; nevertheless, it is indicative of additional futures needed to produce a direct impact on the “enhancing control” factor. The results of this study confirm therapists’ need for additional technical features to improve their limited control over VCP.

### The Video As the Third Agent

This study provides a step toward understanding the complex phenomenon of VCP, offering a new perspective on the reasons behind clinicians’ hesitancy to adopt VCP [10,19,32]. The general system theory (GST) [71] framework was used across the study; this allowed to include the video in the analysis and demonstrate its capacity to affect the VCP as a system. Indeed, the GST substituted the robot model (stimulus-response model) where the rapport and communication involved the psychologist (an observing subject) and the client (an observed object). The GST proposed a new perspective where there is a wider system and within it, there are the 2 interacting agents (psychologist and patient) influencing each other and the whole system. Following this theory, a third interactive agent in the wider system was included, which participates in the relational and communication process.

The video acts as an additional agent and prevents therapists from reaching the other side of the screen or affecting the patient’s reality. The video, as a third party in the therapist-patient interaction, shows that not only does the monitor mediate therapist-patient communication, but it also interferes with the psychotherapy delivery and the development of the therapeutic processes [32]. As a result, therapists perceive poor or no control over the therapy and struggle to build a relationship with clients.

Psychologists’ technical and relational issues were also found in a previous study [47], where they were less prone to use VCP compared to in-person therapy due to the challenges of delivering VCP. However, it may be possible that the challenges mentioned by therapists mirror the intimidation they experienced while transitioning from FTF to VCP [47]. This may explain the inconsistent data showing that therapists and patients have different VCP experiences [32]. Therapists may feel unable to translate their communication and relational therapeutic skills into VCP due to the perceived depersonalizing nature of VCTs [47].

The inability to influence the patient’s environment results in the lack of control over the VCP session and processes [72], which may impact trust development (TA), TR, cognitive and emotional load, and difficulties in connecting emotionally with their clients. Therefore, additional technical features may help therapists establish and maintain better eye contact with their clients (impacting empathy, trust, emotional and cognitive load, and the TR), focus more on clients’ facial expressions by zooming in and out, and reduce distractions caused by popup notifications. With these improvements, clinicians might experience better control over their therapy sessions. Therefore, improved control over VCP would enable therapists to minimize the impact of the video on the therapy session, influence patients’ reality, and facilitate successful therapy outcomes. Although some VC platforms offer features for business purposes [11], there are limited data available that show which VC platforms may better suit psychologists’ needs.

### Which VC Platform for Psychologists?

Companies such as Zoom, Microsoft Teams, and Cisco Webex grew in demand during the COVID-19 pandemic due to the need for remote meetings. According to Pot [73], the top 5 videoconferencing platforms in 2022 were Zoom (for sizeable video calls), Google Meet (for workspace users), Microsoft Teams (the best combination of team chat and videoconferencing app), Whereby Web (the best videoconferencing app for user-friendliness), and WebEx Meeting (video quality). However, the Australian Psychology Society suggests the following principles to guide the choice of platform for psychotherapy: (1) a client-centered approach to their choice of technology, (2) VCTs that are fit for purpose, and (3) VCTs that meet privacy obligations. Privacy, ethics, and legal aspects are incumbent areas to be investigated [3,74], as therapists are generally unaware of the laws regulating these matters in their countries [75]. Currently, Doxe.me is HIPAA (Health Insurance Portability and Accountability Act) compliant, which means that users can safely share sensitive information. However, there is no evidence yet suggesting which VC platforms may better suit psychologists’ needs considering the complexity of the VCP phenomenon and all the processes involved.

### Study Limitations

The results reported in this study show that implementing certain technical features into videoconferencing platforms could improve clinicians’ experiences with VCP. The results should be interpreted carefully due to limitations involving convenience sampling, self-reported data, missing data, and the lack of follow-up. It is acknowledged that additional studies may need to be conducted to examine the efficacy of these features. The results should be further validated to verify whether these features lead to real change in VCP clinical outcomes. Additionally, it would be beneficial to cross-validate the findings from the client’s perspective and see if the same factors are identified. The questionnaire can be used in any research related to telehealth or adopted to facilitate any online activity. Furthermore, no weighting of items was applied. Consequently, refining a scoring algorithm by comprising the weight of each item could provide insights into the relative importance of each question/belief.

Moreover, it was particularly challenging to recruit a large number of participants. Because this was an exploratory study with relatively small samples, further research should rely on a larger sample of clinicians, from different countries, with different levels of computer literacy and years of experience, which may affect the development of the therapeutic processes. For example, it is undeniable that the more professionals are familiar with computers, the fewer constraints they will find [76]. Although this study provides important insights into VCP, it also has potential limitations; for instance, no actions were taken to inhibit multiple entries. However, this risk was mitigated by the controlled distribution of the survey to a specific professional target. Additionally, no measures were implemented to exclude answers based on completion time. Moreover, this study’s cross-sectional design prevents it from inferring causality and assessing participants’ behaviors over time. Future studies should rely on larger, more representative samples and take a longitudinal approach. This would enable the detection of cause-and-effect relationships and a more accurate measurement of the data collected. In addition, future studies should validate the model’s usability, clinicians’ responses to the introduction of the new features, and clinicians’ perceptions of the outcomes derived from using the features. Furthermore, this study did not consider potential technical challenges, feasibility, privacy concerns, technological costs, and ethical considerations, which all deserve a specific focus, as many therapists articulated these concerns [47].

### Conclusion

The findings emphasize that VCP limits therapists’ ability to influence therapy due to technical and relational challenges, leading to a perceived lack of control over the therapeutic process. This study highlights that the video interface imposes specific constraints that challenge the therapist's ability to fully engage with clients. Positioned between the therapist and the patient, the video prevents therapists from influencing the patient’s reality. From this perspective, the video acts as a third agent in the communication and relational system, and it may interfere with the session delivery and the treatment outcome. Consequently, therapists must work around the influence of the third agent (the video), which is absent in traditional in-person consultations. This contributes to their resistance and reluctance toward VCTs. Without additional features, concerns about control will persist.

Understanding clinicians’ technical needs is necessary for creating additional VCT functionalities and improving the VCP experience. By implementing supportive technical features, therapists may gain greater control over therapy, reducing their hesitancy toward VCP and increasing its adoption. Moreover, technical improvements could enhance the VCP experience for patients in remote areas, positioning VCP as a valid alternative to traditional in-person sessions.

This study provides insights into telemedicine, driven by technological advancements and evolutions in medical settings. It highlights the need for professionals to establish effective communication and relationships with their target populations. Future research should focus on the role of the computer in the relationship-building process between the clinician and client. This might lead to the possible development of further technological features and interfaces. Moreover, it is pivotal to identify feasible, practical features and assess their efficacy in improving psychologists’ VCP experience. It will be necessary to test these technical features in different clinical settings and therapeutic approaches.

To further validate the theoretical model, more data related to usability and applicability are needed. Data should be collected across different clinical specialties and different patient cohorts, including age (as some groups might be more proficient technology users than others) and different diagnostic groups (as motor and cognitive aspects of the use are crucial). The relationship between clinicians’ demographics (ie, age, years in practice, and level of computer/technology literacy) and their responses to survey questions should also be assessed.

Finally, because the results show the need for further technical features as the volume of challenges that clinicians face grows, more features are needed to support clinicians in building a better relationship with their clients and maintaining greater control over the VCP system. It is crucial for clinicians to exert a meaningful real impact on the therapeutic process. Future studies should explore the privacy and ethical concerns arising from the use of VCTs.

## Appendix

Multimedia Appendix 1 Table S1. Survey distributed to the recruited sample.

Multimedia Appendix 2 Table S2. Correlation matrix: relation analysis among items.

## Abbreviations

Adjβ: adjusted beta
EFA: exploratory factorial analysis
FTF: face-to-face
GST: general system theory
HIPAA: Health Insurance Portability and Accountability Act
TA: therapeutic alliance
TR: therapeutic relationship
VCP: videoconference psychotherapy
VCT: videoconferencing technology

## Data Availability

The data collected and analyzed during this study were deidentified and are available from the corresponding author upon reasonable request.
