# Supplementary material for: Exploring Technical Features to Enhance Control in Videoconferencing Psychotherapy: Quantitative Study on Clinicians’ Perspectives
Source: J Med Internet Res. 2025 Apr 1;27:e66904. doi: 10.2196/66904 (PMC12000784; doi:10.2196/66904)
Supplement: Multimedia Appendix 1 [file jmir_v27i1e66904_app1.docx]

Table 1. Survey distributed to the sample recruited.

| Variables | Items | Reference |
| --- | --- | --- |
| **Attitude** | Item 20  My emotional and cognitive preparation before sessions varies depending on whether the encounter is online or face-to-face | Cataldo, F., et al., Enhancing Therapeutic Processes in Videoconferencing Psychotherapy: Interview Study of Psychologists’ Technological Perspective. JMIR Formative Research, 2023. |
|  | Item 23  My attitude toward videoconference psychotherapy is positively changing. | Connolly SL, Miller CJ, Lindsay JA, Bauer MS. A systematic review of providers' attitudes toward telemental health via videoconferencing. Clin Psychol (New York) 2020 Jan 06;27(2):e12311.  Cataldo, F., et al., Enhancing Therapeutic Processes in Videoconferencing Psychotherapy: Interview Study of Psychologists’ Technological Perspective. JMIR Formative Research, 2023. |
|  | Item 24  I think I will keep using videoconference platforms to treat my clients in the future. | Cataldo, F., et al., Enhancing Therapeutic Processes in Videoconferencing Psychotherapy: Interview Study of Psychologists’ Technological Perspective. JMIR Formative Research, 2023. |
| **Presence** | Item 1  Eye contact functionality (maintaining and establishing eye contact) would help me to reassure patients of my presence. | Wilson JM, Straus SG, McEvily B. All in due time: the development of trust in computer-mediated and face-to-face teams. Organizational Behavior and Human Decision Processes 2006 Jan;99(1):16-33;  Cataldo, F., et al., Enhancing Therapeutic Processes in Videoconferencing Psychotherapy: Interview Study of Psychologists’ Technological Perspective. JMIR Formative Research, 2023. |
|  | Item 6  The technical ability to limit client's online activities during sessions would boost my sense of presence with clients. | Cataldo, F., et al., Enhancing Therapeutic Processes in Videoconferencing Psychotherapy: Interview Study of Psychologists’ Technological Perspective. JMIR Formative Research, 2023. |
|  | Item 7  Limiting my clients' online activities (e-mails, notifications, browsing online etc.) during our sessions would give me more control over our relationship. | Cataldo, F., et al., Enhancing Therapeutic Processes in Videoconferencing Psychotherapy: Interview Study of Psychologists’ Technological Perspective. JMIR Formative Research, 2023. |
| **Trust** | Item 2  I would improve trust if I could establish and maintain better eye contact with my clients during video sessions. | Bos, N., Olson, J., Gergle, D., Olson, G., & Wright, Z. (2002). Effects of four computer-mediated communications channels on trust development. Paper presented at the Proceedings of the SIGCHI conference on human factors in computing systems.  Wilson JM, Straus SG, McEvily B. All in due time: the development of trust in computer-mediated and face-to-face teams. Organizational Behav Human Decision Process 2006 Jan;99(1):16-33.  Newcomb AB, Duval M, Bachman SL, Mohess D, Dort J, Kapadia MR. Building rapport and earning the surgical patient's trust in the era of social distancing: teaching patient-centered communication during video conference encounters to medical students. J Surg Educ 2021 Jan;78(1):336-341.  Cataldo, F., et al., Enhancing Therapeutic Processes in Videoconferencing Psychotherapy: Interview Study of Psychologists’ Technological Perspective. JMIR Formative Research, 2023. |
|  | Item 12  I struggle to build trust with my clients by Video. | Nardi, B., & Whittaker, S. (2002). The place of face-to-face communication in distributed work. 83-110.  Rees CS, Stone S. Therapeutic alliance in face-to-face versus video conferenced psychotherapy. Professional Psychology: Research and Practice 2005;36(6):649-653.  Fletcher-Tomenius LJ, Vossler A. Trust in online therapeutic relationships: the therapist's experience. Counsel Psychol Rev 2009 Mar 01;24(2):24-33.  Newcomb AB, Duval M, Bachman SL, Mohess D, Dort J, Kapadia MR. Building rapport and earning the surgical patient's trust in the era of social distancing: teaching patient-centered communication during video conference encounters to medical students. J Surg Educ 2021 Jan;78(1):336-341.  Cataldo, F., et al., Enhancing Therapeutic Processes in Videoconferencing Psychotherapy: Interview Study of Psychologists’ Technological Perspective. JMIR Formative Research, 2023. |
|  | Item 13  I would use a phone call before the first video session to increase trust with my clients. | Cataldo, F., et al., Enhancing Therapeutic Processes in Videoconferencing Psychotherapy: Interview Study of Psychologists’ Technological Perspective. JMIR Formative Research, 2023. |
| **Engagement** | Item 3  I feel I need to establish eye contact to reinforce my engagement with clients during my video sessions. | Wilson JM, Straus SG, McEvily B. All in due time: the development of trust in computer mediated and face-to-face teams. Organizational Behavior and Human Decision Processes 2006 Jan;99(1):16-33.  Newcomb AB, Duval M, Bachman SL, Mohess D, Dort J, Kapadia MR. Building rapport and earning the surgical patient's trust in the era of social distancing: teaching patient-centered communication during video conference encounters to medical students. J Surg Educ 2021 Jan;78(1):336-341.  Cataldo, F., et al., Enhancing Therapeutic Processes in Videoconferencing Psychotherapy: Interview Study of Psychologists’ Technological Perspective. JMIR Formative Research, 2023. |
|  | Item 4  The eye contact functionality (establishing-maintaining eye contact) would heighten my chances to empathise with clients. | Terry C, Cain J. The emerging issue of digital empathy. Am J Pharm Educ 2016 May 25;80(4):58.  Hall JA, Harrigan JA, Rosenthal R. Nonverbal behavior in clinician—patient interaction. Appl Preventive Psychol 1995 Dec;4(1):21-37.  Chen M. Leveraging the asymmetric sensitivity of eye contact for videoconference. In: Proceedings of the SIGCHI. Conference on Human Factors in Computing Systems. 2002 Presented at: CHI02: Human Factors in Computing Systems; Apr 20 - 25, 2002; Minneapolis Minnesota USA.  Tam T, Cafazzo JA, Seto E, Salenieks ME, Rossos PG. Perception of eye contact in video teleconsultation. J Telemed Telecare 2007 Jun 24;13(1):35-39.  Grondin F, Lomanowska AM, Békés V, Jackson PL. A methodology to improve eye contact in telepsychotherapy via videoconferencing with considerations for psychological distance. Counsel Psychol Q 2020 Jun 21;34(3-4):586-599. |
|  | Item 8  Limiting my clients’ online activities (e-mails, notifications, browsing online etc) during our sessions would support me in enhancing my engagement with clients. | Cataldo, F., et al., Enhancing Therapeutic Processes in Videoconferencing Psychotherapy: Interview Study of Psychologists’ Technological Perspective. JMIR Formative Research, 2023. |
|  | Item 9  I feel that zooming In/Out (focus on clients’ facial expressions/whole body) would augment my level of engagement with clients. | Cataldo, F., et al., Enhancing Therapeutic Processes in Videoconferencing Psychotherapy: Interview Study of Psychologists’ Technological Perspective. JMIR Formative Research, 2023. |
|  | Item 10  Zooming In/Out (focus on clients’ facial expressions/whole body) would allow me to understand clients’ emotions. | Cataldo, F., et al., Enhancing Therapeutic Processes in Videoconferencing Psychotherapy: Interview Study of Psychologists’ Technological Perspective. JMIR Formative Research, 2023. |
| **Cognitive and Emotional load** | Item 5  My cognitive and emotional load would be reduced if I could maintain better eye contact with clients during video sessions. | Fosslien, L., & Duffy, M. W. (2020). How to combat zoom fatigue. Harvard Business Review.    Cataldo, F., et al., Enhancing Therapeutic Processes in Videoconferencing Psychotherapy: Interview Study of Psychologists’ Technological Perspective. JMIR Formative Research, 2023. |
|  | Item 11  To reduce my cognitive and emotional load I need a platform that allows me to focus on clients’ facial expressions (zooming in) and capture the whole body (zooming out). | Cataldo, F., et al., Enhancing Therapeutic Processes in Videoconferencing Psychotherapy: Interview Study of Psychologists’ Technological Perspective. JMIR Formative Research, 2023. |
|  | Item 22  My fatigue would be reduced if my telehealth platform enabled simultaneous interaction with other apps and software. | Fosslien, L., & Duffy, M. W. (2020). How to combat zoom fatigue. Harvard Business Review.  Cataldo, F., et al., Enhancing Therapeutic Processes in Videoconferencing Psychotherapy: Interview Study of Psychologists’ Technological Perspective. JMIR Formative Research, 2023. |
| **Therapeutic Relationship** | Item 14  I find it difficult to build a therapeutic relationship with my clients by video. | Riva G. Is presence a technology issue? Some insights from cognitive sciences. Virtual Reality 2009 May 24;13(3):159-169.  Fletcher-Tomenius LJ, Vossler A. Trust in online therapeutic relationships: the therapist's experience. Counsel Psychol  Rev 2009 Mar 01;24(2):24-33.  Connolly SL, Miller CJ, Lindsay JA, Bauer MS. A systematic review of providers' attitudes toward telemental health via videoconferencing. Clin Psychol (New York) 2020 Jan 06;27(2):e12311.  Cataldo, F., et al., A perspective on client-psychologist relationships in videoconferencing psychotherapy: Literature review. JMIR mental health, 2021.  Cataldo F, Chang S, Mendoza A, Buchanan G. A perspective on client-psychologist relationships in videoconferencing psychotherapy: literature review. JMIR Ment Health 2021 Feb 19;8(2):e19004 |
|  | Item 15  I feel I have less control over my therapeutic relationship via video rather than face to face. | Rees CS, Stone S. Therapeutic alliance in face-to-face versus videoconferenced psychotherapy. Professional Psychol Res Pract 2005 Dec;36(6):649-653.  Fletcher-Tomenius LJ, Vossler A. Trust in online therapeutic relationships: the therapist's experience. Counsel Psychol Rev 2009 Mar 01;24(2):24-33.  Connolly SL, Miller CJ, Lindsay JA, Bauer MS. A systematic review of providers' attitudes toward telemental health via videoconferencing. Clin Psychol (New York) 2020 Jan 06;27(2):e12311.  Cataldo, F., et al., A perspective on client-psychologist relationships in videoconferencing psychotherapy: Literature review. JMIR mental health, 2021. |
| **Video Communication** | Item 16  I perceive the monitor as an additional member of the therapeutic interaction. | Cataldo, F., et al., A perspective on client-psychologist relationships in videoconferencing psychotherapy: Literature review. JMIR mental health, 2021.  Cataldo, F., et al., Enhancing Therapeutic Processes in Videoconferencing Psychotherapy: Interview Study of Psychologists’ Technological Perspective. JMIR Formative Research, 2023. |
|  | Item 17  I struggle to communicate by video with my clients. | Cataldo, F., et al., A perspective on client-psychologist relationships in videoconferencing psychotherapy: Literature review. JMIR mental health, 2021. |
|  | Item 18  I feel the video hinders my psychotherapy sessions. | Cataldo, F., et al., A perspective on client-psychologist relationships in videoconferencing psychotherapy: Literature review. JMIR mental health, 2021.  Omodei M, McClennan J. Technology in Mental Health: Applications in Practice, Supervision and Training. Springfield, USA: Charles C Thomas Publisher; 2016.  Rees CS, Stone S. Therapeutic alliance in face-to-face versus videoconferenced psychotherapy. Professional Psychology: Research and Practice 2005;36(6):649-653. |
|  | Item 19  I believe it is hard to connect emotionally with my clients through video. | Wray B, Rees C. Is there a role for videoconferencing in cognitive behavioural therapy. Paper presented at the 11th Australian Association for Cognitive and Behaviour Therapy State Conference, Perth, WA, Australia 2003.  Cataldo, F., et al., Enhancing Therapeutic Processes in Videoconferencing Psychotherapy: Interview Study of Psychologists’ Technological Perspective. JMIR Formative Research, 2023. |
| **Safety** | Item 21  I would feel safe during my video sessions by having an emergency call functionality to connect my problematic clients with hospitals, police etc. | Cataldo, F., et al., Enhancing Therapeutic Processes in Videoconferencing Psychotherapy: Interview Study of Psychologists’ Technological Perspective. JMIR Formative Research, 2023. |
