# Supplementary material for: Exploring Technical Features to Enhance Control in Videoconferencing Psychotherapy: Quantitative Study on Clinicians’ Perspectives
Source: J Med Internet Res. 2025 Apr 1;27:e66904. doi: 10.2196/66904 (PMC12000784; doi:10.2196/66904)
Supplement: Multimedia Appendix 2 [file jmir_v27i1e66904_app2.docx]

Table 2. Correlation Matrix: relation analysis among items.

|  | 1 | 2 | 3 | 4 | 5 | 6 | 7 | 8 | 9 | 10 | 11 | 12 | 13 | 14 | 15 | 16 | 17 | 18 | 19 | 20 | 21 | 22 | 23 | 24 |
| --- | --- | --- | --- | --- | --- | --- | --- | --- | --- | --- | --- | --- | --- | --- | --- | --- | --- | --- | --- | --- | --- | --- | --- | --- |
| 1. Eye contact functionality (maintaining and establishing eye contact) would help me to reassure patients of my presence. | 1 |  |  |  |  |  |  |  |  |  |  |  |  |  |  |  |  |  |  |  |  |  |  |  |
| 2. I would improve trust if I could establish and maintain better eye contact with my clients during video sessions | 0.43, 0.009 | 1 |  |  |  |  |  |  |  |  |  |  |  |  |  |  |  |  |  |  |  |  |  |  |
| 3. I feel I need to establish eye contact to reinforce my engagement with clients during my video sessions. | 0.61, <0.001 | 0.55, <0.001 | 1 |  |  |  |  |  |  |  |  |  |  |  |  |  |  |  |  |  |  |  |  |  |
| 4. The eye contact functionality (establishing-maintaining eye contact) would heighten my chances to empathise with clients. | 0.46, 0.002 | 0.53, <0.001 | 0.65, <0.001 | 1 |  |  |  |  |  |  |  |  |  |  |  |  |  |  |  |  |  |  |  |  |
| 5. My cognitive and emotional load would be reduced if I could maintain better eye contact with clients during video sessions. | 0.36, 0.191 | 0.57, <0.001 | 0.49, 0.001 | 0.60, <0.11 | 1 |  |  |  |  |  |  |  |  |  |  |  |  |  |  |  |  |  |  |  |
| 6. The technical ability to limit client's online activities during sessions would boost my sense of presence with clients. | 0.40, 0.039 | 0.40, 0.035 | 0.43, 0.010 | 0.42, 0.018 | 0.37, 0.112 | 1 |  |  |  |  |  |  |  |  |  |  |  |  |  |  |  |  |  |  |
| 7. Limiting my clients' online activities (e-mails, notifications, browsing online etc.) during our sessions would give me more control over our relationship. | 0.39, 0.055 | 0.41, 0.022 | 0.35, 0.296 | 0.27, 1.000 | 0.31, 1.000 | 0.74, <0.001 | 1 |  |  |  |  |  |  |  |  |  |  |  |  |  |  |  |  |  |
| 8. Limiting my clients’ online activities (e-mails, notifications, browsing online etc) during our sessions would support me in enhancing my engagement with clients. | 0.23, 1.000 | 0.32, 0.838 | 0.24, 1.000 | 0.31, 0.987 | 0.28, 1.000 | 0.70, <0.001 | 0.77, <0.001 | 1 |  |  |  |  |  |  |  |  |  |  |  |  |  |  |  |  |
| 9. I feel that zooming In/Out (focus on clients’ facial expressions/whole body) would augment my level of engagement with clients. | 0.36, 0.166 | 0.45, 0.003 | 0.40, 0.046 | 0.42, 0.020 | 0.46, 0.002 | 0.27, 1.000 | 0.30, 1.000 | 0.31, 1.000 | 1 |  |  |  |  |  |  |  |  |  |  |  |  |  |  |  |
| 10. Zooming In/Out (focus on clients’ facial expressions/whole body) would allow me to understand clients’ emotions. | 0.36, 0.188 | 0.47, 0.001 | 0.37, 0.136 | 0.46, 0.002 | 0.49, 0.001 | 0.28, 1.000 | 0.31, 0.895 | 0.32, 0.742 | 0.80, <0.001 | 1 |  |  |  |  |  |  |  |  |  |  |  |  |  |  |
| 11. To reduce my cognitive and emotional load I need a platform that allows me to focus on clients’ facial expressions (zooming in) and capture the whole body (zooming out). | 0.32, 0.680 | 0.42, 0.015 | 0.41, 0.022 | 0.53, <0.001 | 0.59, <0.001 | 0.33, 0.59 | 0.37, 0.120 | 0.33, 0.622 | 0.67, <0.001 | 0.62, <0.001 | 1 |  |  |  |  |  |  |  |  |  |  |  |  |  |
| 12. I struggle to build trust with my clients by Video. | 0.20, 1.000 | 0.26, 1.000 | 0.12, 1.000 | 0.21, 1.000 | 0.24, 1.000 | 0.03, 1.000 | 0.14, 1.000 | 0.04, 1.000 | 0.24, 1.000 | 0.19, 1.000 | 0.30, 1.000 | 1 |  |  |  |  |  |  |  |  |  |  |  |  |
| 13. I would use a phone call before the first video session to increase trust with my clients. | -0.03, 1.000 | 0.13, 1.000 | 0.06, 1.000 | 0.20, 1.000 | 0.01, 1.000 | 0.02, 1.000 | -0.04, 1.000 | 0.03, 1.000 | 0.05, 1.000 | 0.04, 1.000 | 0.148, 1.000 | 0.17, 1.000 | 1 |  |  |  |  |  |  |  |  |  |  |  |
| 14. I find it difficult to build a therapeutic relationship with my clients by video. | 0.20, 1.000 | 0.34, 0.429 | 0.13, 1.000 | 0.11, 1.000 | 0.22, 1.000 | 0.06, 1.000 | 0.13, 1.000 | 0.02, 1.000 | 0.23, 1.000 | 0.18, 1.000 | 0.22, 1.000 | 0.80, <0.001 | 0.06, 1.000 | 1 |  |  |  |  |  |  |  |  |  |  |
| 15. I feel I have less control over my therapeutic relationship via video rather than face to face. | 0.33, 0.484 | 0.41, 0.023 | 0.22, 1.000 | 0.26, 1.000 | 0.30, 1.000 | 0.17, 1.000 | 0.15, 1.000 | 0.12, 1.000 | 0.27, 1.000 | 0.22, 1.000 | 0.26, 1.000 | 0.61, <0.001 | 0.34, 0.405 | 0.67, <0.001 | 1 |  |  |  |  |  |  |  |  |  |
| 16. I perceive the monitor as an additional member of the therapeutic interaction. | 0.08, 1.000 | 0.18, 1.000 | 0.31, 0.896 | 0.22, 1.000 | 0.15, 1.000 | 0.30, 1.000 | 0.27, 1.000 | 0.28, 1.000 | 0.14, 1.000 | 0.14, 1.000 | 0.16, 1.000 | -0.03, 1.000 | 0.16, 1.000 | 0.01, 1.000 | 0.12, 1.000 | 1 |  |  |  |  |  |  |  |  |
| 17. I struggle to communicate by video with my clients. | 0.15, 1.000 | 0.26, 1.000 | 0.09, 1.000 | 0.18, 1.000 | 0.20, 1.000 | 0.05, 1.000 | 0.15, 1.000 | 0.003, 1.000 | 0.18, 1.000 | 0.16, 1.000 | 0.22, 1.000 | 0.79, <0.001 | 0.13, 1.000 | 0.83, <0.001 | 0.57, <0.001 | 0.06, 1.000 | 1 |  |  |  |  |  |  |  |
| 18. I feel the video hinders my psychotherapy sessions. | 0.23, 1.000 | 0.245, 1.000 | 0.17, 1.000 | 0.15, 1.000 | 0.15, 1.000 | 0.10, 1.000 | 0.15, 1.000 | 0.01, 1.000 | 0.18, 1.000 | 0.09, 1.000 | 0.19, 1.000 | 0.72, <0.001 | 0.18, 1.000 | 0.73, <0.001 | 0.72, <0.001 | 0.05, 1.000 | 0.76, <0.001 | 1 |  |  |  |  |  |  |
| 19. I believe it is hard to connect emotionally with my clients through video. | 0.29, 1.000 | 0.36, 0.173 | 0.24, 1.000 | 0.247, 1.000 | 0.22, 1.000 | 0.07, 1.000 | 0.12, 1.000 | 0.04, 1.000 | 0.32, 0.837 | 0.25, 1.000 | 0.247, 1.000 | 0.74, <0.001 | 0.18, 1.000 | 0.82, <0.001 | 0.69, <0.001 | 0.10, 1.000 | 0.71, <0.001 | 0.70, <0.001 | 1 |  |  |  |  |  |
| 20. My emotional and cognitive preparation before sessions varies depending on whether the encounter is online or face-to-face | 0.0047, 1.000 | 0.29, 1.000 | 0.09, 1.000 | 0.10, 1.000 | 0.14, 1.000 | -0.003, 1.000 | -0.005, 1.000 | 0.03, 1.000 | 0.14, 1.000 | 0.16, 1.000 | 0.07, 1.000 | 0.45, 0.003 | 0.08, 1.000 | 0.54, <0.001 | 0.54, <0.001 | 0.12, 1.000 | 0.41, 0.024 | 0.48, 0.001 | 0.51, <0.001 | 1 |  |  |  |  |
| 21. I would feel safe during my video sessions by having an emergency call functionality to connect my problematic clients with hospitals, police etc. | 0.02, 1.000 | 0.04, 1.000 | 0.17, 1.000 | 0.22, 1.000 | 0.20, 1.000 | 0.16, 1.000 | 0.17, 1.000 | 0.23, 1.000 | 0.13, 1.000 | 0.21, 1.000 | 0.38, 0.073 | -0.004, 1.000 | -0.04, 1.000 | -0.07, 1.000 | -0.11, 1.000 | -0.12, 1.000 | -0.08, 1.000 | -0.05, 1.000 | -0.08, 1.000 | 0.12, 1.000 | 1 |  |  |  |
| 22. My fatigue would be reduced if my telehealth platform enabled simultaneous interaction with other apps and software. | -0.04, 1.000 | 0.28, 1.000 | 0.06, 1.000 | 0.18, 1.000 | 0.36, 0.278 | 0.13, 1.000 | 0.02, 1.000 | 0.08, 1.000 | 0.349, 0.278 | 0.30, 1.000 | 0.40, 0.037 | 0.06, 1.000 | 0.02, 1.000 | 0.15, 1.000 | 0.02, 1.000 | 0.12, 1.000 | 0.148, 1.000 | -0.01, 1.000 | 0.01, 1.000 | 0.12, 1.000 | 0.43, 0.012 | 1 |  |  |
| 23. My attitude toward videoconference psychotherapy is positively changing. | -0.10, 1.000 | 0.07, 1.000 | 0.03, 1.000 | 0.08, 1.000 | 0.09, 1.000 | 0.07, 1.000 | 0.003, 1.000 | 0.19, 1.000 | -0.002, 1.000 | 0.14, 1.000 | -0.16, 1.000 | -0.38, 0.071 | -0.26, 1.000 | -0.40, 0.037 | -0.42, 0.0145 | 0.03, 1.000 | -0.45  ,0.003 | -0.47, 0.001 | -0.40, 0.034 | -0.09, 1.000 | 0.22, 1.000 | 0.24, 1.000 | 1 |  |
| 24. I think I will keep using videoconference platforms to treat my clients in the future | -0.16, 1.000 | -0.22, 1.000 | -0.08, 1.000 | 0.03, 1.000 | -0.12, 1.000 | -0.08, 1.000 | -0.10, 1.000 | 0.02, 1.000 | -0.004, 1.000 | 0.02, 1.000 | -0.02, 1.000 | -0.62, <0.001 | -0.20, 1.000 | -0.646, <0.001 | -0.53, <0.001 | -0.14, 1.000 | -0.61, <0.001 | -0.60, <0.001 | -0.57, <0.001 | -0.32, 0.661 | 0.28, 1.000 | 0.08, 1.000 | 0.47, 0.002 | 1 |
